# Statins but Not Aspirin Reduce Thrombotic Risk Assessed by Thrombin Generation in Diabetic Patients without Cardiovascular Events: The RATIONAL Trial

**DOI:** 10.1371/journal.pone.0032894

**Published:** 2012-03-28

**Authors:** Alejandro Macchia, Nicolás Laffaye, Pablo D. Comignani, Elena Cornejo Pucci, Cecilia Igarzabal, Alejandra S. Scazziota, Lourdes Herrera, Javier A. Mariani, Julio C. Bragagnolo, Hugo Catalano, Gianni Tognoni, Antonio Nicolucci

**Affiliations:** 1 Department of Medicine, Hospital Alemán, Buenos Aires, Argentina; 2 Department of Medicine, Centro de Educación Médica e Investigaciones Clínicas (CEMIC), Buenos Aires, Argentina; 3 Department of Clinical Pharmacology and Epidemiology, Consorzio Mario Negri Sud, Santa Maria Imbaro, Chieti, Italy; 4 Fundación GESICA (Grupo de Estudio Sobre Investigación Clínica en Argentina), Buenos Aires, Argentina; 5 Facultad de Farmacia y Bioquímica, Universidad de Buenos Aires, Buenos Aires, Argentina; 6 Department of Medicine, Hospital Ramos Mejía, Buenos Aires, Argentina; Universita Magna-Graecia di Catanzaro, Italy

## Abstract

**Background:**

The systematic use of aspirin and statins in patients with diabetes and no previous cardiovascular events is controversial. We sought to assess the effects of aspirin and statins on the thrombotic risk assessed by thrombin generation (TG) among patients with type II diabetes mellitus and no previous cardiovascular events.

**Methodology/Principal Findings:**

Prospective, randomized, open, blinded to events evaluation, controlled, 2×2 factorial clinical trial including 30 patients randomly allocated to aspirin 100 mg/d, atorvastatin 40 mg/d, both or none. Outcome measurements included changes in TG levels after treatment (8 to 10 weeks), assessed by a calibrated automated thrombogram. At baseline all groups had similar clinical and biochemical profiles, including TG levels. There was no interaction between aspirin and atorvastatin. Atorvastatin significantly reduced TG measured as peak TG with saline (85.09±55.34 nmol vs 153.26±75.55 nmol for atorvastatin and control groups, respectively; p = 0.018). On the other hand, aspirin had no effect on TG (121.51±81.83 nmol vs 116.85±67.66 nmol, for aspirin and control groups, respectively; p = 0.716). The effects of treatments on measurements of TG using other agonists were consistent.

**Conclusions/Significance:**

While waiting for data from ongoing large clinical randomized trials to definitively outline the role of aspirin in primary prevention, our study shows that among diabetic patients without previous vascular events, statins but not aspirin reduce thrombotic risk assessed by TG.

**Trial Registration:**

ClinicalTrials.gov NCT00793754

## Introduction

Individuals with type 2 diabetes have a two- to four-fold increased risk of cardiovascular disease (CVD) compared with non-diabetic subjects [1]. Since it has been shown that diabetic patients without previous MI have as high risk of events as non-diabetic patients with previous MI, [2] diabetes could be considered as a coronary equivalent, and suggests the need for treating cardiovascular risk factors aggressively.

Evidence supporting efficacy of preventive strategies in individuals with diabetes is surprisingly scant. In particular, recommendations [3] regarding the use of aspirin for the prevention of cardiovascular events in patients with diabetes reflect an extrapolation of data derived from other high risk populations, rather than reliable trial-based evidence in individuals with diabetes [4]. Systematic reviews [5], [6] and clinical trials [7], [8] do not fully support the universal recommendation of aspirin in patients with diabetes. The evidence failed to show an appropriate balance for patients with diabetes between the small/neutral protective effects of aspirin and the constant risk of bleeding. In fact, physicians seem to be reluctant to follow guidelines, as a very low proportion of patients with diabetes are treated with aspirin for the prevention of cardiovascular events [9]. On the other hand, statins had been proven effective for primary prevention in populations with known moderate to high risk of cardiovascular events [10], [11]. This recommendation is equally valid for patients with and without diabetes [10], [11].

It is currently debated if people with diabetes but without previous cardiovascular events should be universally prescribed with aspirin, statins or a combination of both [12]. The aim of the present study was to assess the effect of aspirin, statins or their combination on thrombotic risk assessed by monitoring thrombin generation (TG), a valid and reliable method that allows description of all the phases of TG process, providing a global picture of the thrombotic risk as well as the influence/efficacy of anti-thrombotic strategies [13]–[16].

## Methods

The protocol for this trial and supporting CONSORT checklist are available as supporting information; see Checklist S1 and Protocol S1.

The RATIONAL (aspiRin stAtins or boTh for the reductIon of thrOmbin geNeration in diAbetic peopLe – clinical trial identifier NCT00793754) study was a prospective, randomized, open, and blinded to evaluations, factorial 2×2 clinical trial conducted at public and private hospitals in Buenos Aires, Argentina.

### Participants

**Figure 1:**
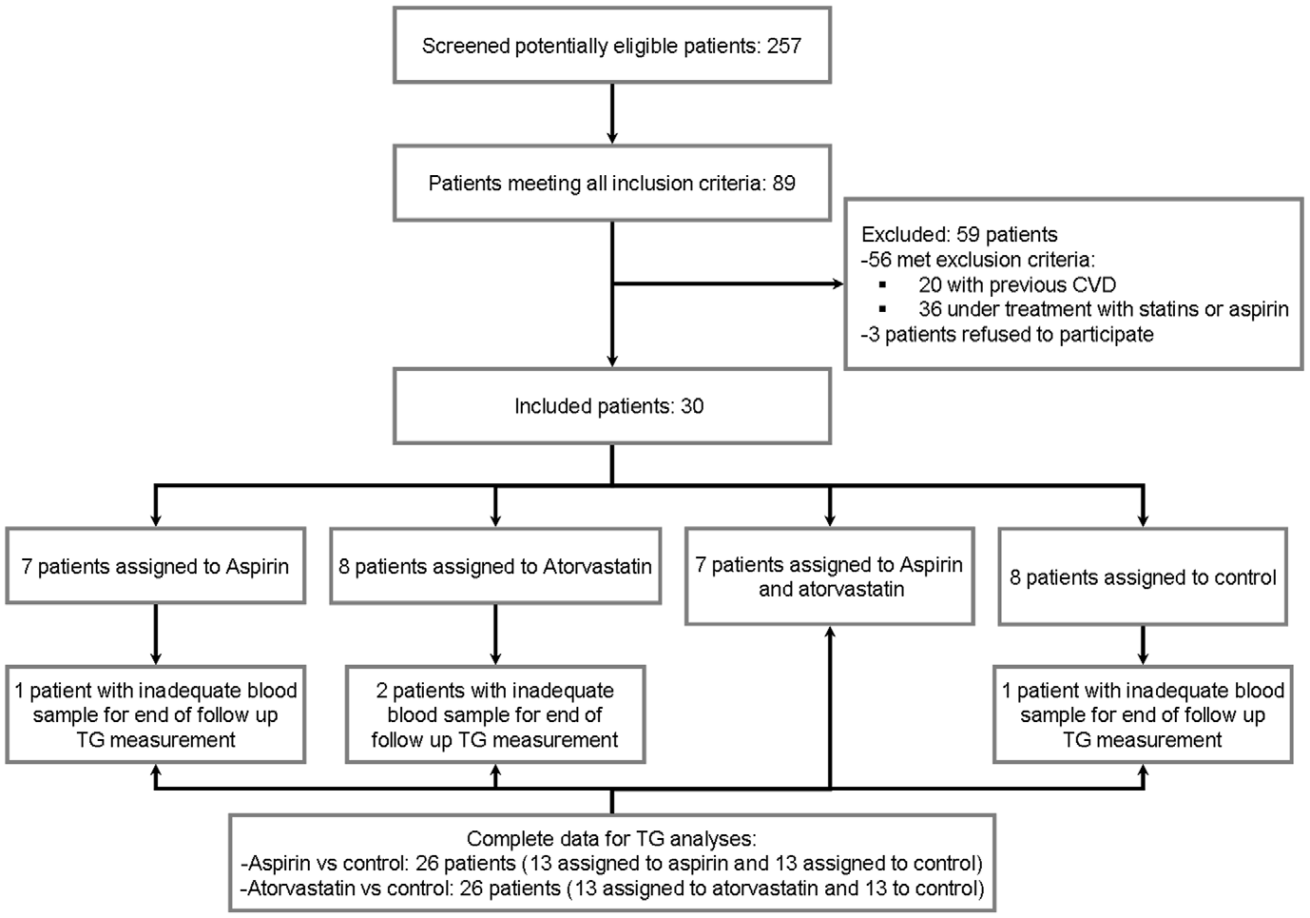
Study flowchart.

All patients were consecutively recruited from outpatient clinics specialized in internal medicine or diabetology where they were under care. Patients were males or females aged ≥50 years diagnosed with type 2 diabetes based on standard criteria [17] at least 1 year prior to study entry. Inclusion criteria included treatment for diabetes with either oral agents or insulin therapy for at least the past one year, no previous cardiovascular events, and no treatment with aspirin or statins during the year prior to recruitment. The main exclusion criteria were current treatment with aspirin or any antiplatelet agent including sporadic use of NSAID and the presence of previous vascular events or any known hemorrhagic condition (figure 1). At baseline, history of vascular disease and cardiovascular risk factors were assessed during personal interview and in all cases a 12-lead electrocardiogram was performed. All patients who reported previous hospitalized or non hospitalized vascular events were excluded. Abnormal electrocardiogram suggestive of coronary heart disease and patients with left bundle branch block (LBBB) were also excluded.

### Ethics

The IRB committees of CEMIC (centro de educación médica e investigaciones clínicas) and Hospital Alemán approved the study protocol (Protocol S1), and all patients provided signed informed consent before recruitment. The study was conducted in accordance with the Declaration of Helsinki and Good Clinical Practice guidelines.

### Interventions and Randomization procedures

After providing signed informed consent all patients were centrally randomized using a random allocation sequence generated by computer. To maintain allocation concealment, randomization was conducted through central telephone calls to coordinating center during administrative hours in working days. Patients were randomly assigned to one of the following groups: aspirin 100 mg daily; atorvastatin 40 mg daily; aspirin 100 mg plus atorvastatin 40 mg daily, or no treatment for 8 to 10 weeks.

### Objectives

To evaluate the effects of atorvastin and aspirin on TG.

### Baseline evaluations

Before beginning treatment, all patients underwent a complete clinical/demographic and biochemical characterization. Age, sex, weight, height, body mass index (BMI), waist circumference, systolic and diastolic arterial blood pressure were recorded. The presence of relevant conditions including smoking history, familiar antecedents, cardiovascular risk factors (hypertension, dyslipidemia, mother or father with MI before age 60, chronic renal failure, obstructive pulmonary disease, thyroid disorder) and medications were completely surveyed. A complete physical examination was done in all patients including a 12-lead-electrocardiogram. Biochemical determinations included fasting glucose, HbA1c, total cholesterol, HDL and LDL cholesterol, total triglycerides, bilirubin, aspartate aminotransferase, alanine aminotransferase, hemoglobin, hematocrit, white blood cell count, platelets and creatinine.

### TG assessment

To confirm the absence of any drug affecting platelet function, platelet aggregation in plasma-rich platelets (PRP) was measured photometrically in a double-channel Lumi-Aggregometer (Chrono-log Corp., Havertown, PA,USA). The technique, material used and preparation of plasma are described elsewhere [18]. Briefly, in all patients venous blood was withdrawn from the antecubital vein without stasis and mixed with 0.11 mol/L sodium citrate (1∶10 v/v). The PRP was obtained by centrifugation at 900 rpm for 10 minutes at room temperature and platelet poor plasma (PPP) was obtained by centrifuging PRP at 4000 rpm for 10 minutes. PRP was adjusted to a platelet count of 290,000/µL to 310,000/µL with autologous PPP. If contamination of PRP with erythrocytes or leukocytes was observed by light microscopy, a second centrifugation at 900 rpm for 5 minutes was conducted to minimize the number of these cells. Plastic syringes, tubes, and pipettes were used for all tests. TG was assayed by the calibrated automated thrombography technique essentially as described by Hemker et al [19].

### Definitions used

Lag-time (LT): the time in minutes from the start of the assay to the initial generation of thrombin (the moment at which 10 nM thrombin is formed). Time to peak of TG (TTP): the time in minutes required to reach maximum TG. Peak of TG (PTG): the maximum thrombin concentration expressed in nmol/L. Endogenous Thrombin Potential (ETP): area under the curve (AUC) expressed in nmol/L of thrombin.

### End of study evaluations

After a minimum of 8 and a maximum of 10 weeks of treatment, all randomized patients repeated all biochemical and TG measurements.

### Outcomes

Primary end point was the level of TG at the end of follow up, as measured by the peak of TG with saline as agonist. Other outcomes were peak TG with arachidonic acid, tissue factor, ADP and lag time with arachidonic acid.

### Sample size

We calculated that a total of 7 patients in every treatment combination would provide 90% power to detect a difference of at least 55 nmol/l (with an standard deviation of 35 nmol/l) in the assessment of the PTG with saline between groups using a factorial design [18]. This sample size would also provide 80% power to detect the presence of interaction.

### Statistical analysis

To assess the effects of the treatments on the outcomes measures, we used ANOVA for factorial designs. The statistical model included two fixed factors (both treatments) and an interaction term. Under this model, the evaluation of individual treatment effects (main effects) is valid when there is no interaction between treatments (i.e. the effects of one treatment is not influenced by the other treatment). Interaction was evaluated using the interaction term in the ANOVA model and by visual assessment of profile plots. Normality assumption was assessed through visual evaluation of quantil-quantil plots and Shapiro-Wilk's test (in the case of the ANOVA model assumptions, we also used these methods on studentised residuals). The equality of variances assumption was evaluated through the Levene's test.

As no interaction between aspirin and statins was detected, results are presented as a factorial design (e.g. all patients who received aspirin versus those not receiving aspirin; and all patients receiving statins versus those not receiving statins).

Continuous data are presented as means and standard deviations (SD) or medians and interquartile ranges (IQR). Categorical data are described as numbers and percentages. To compare groups regarding baseline continuous data, we used Student's T test or the Mann-Whitney U test for normally or not normally distributed data, respectively. Categorical data were compared using the Exact Fisher's test. All analyses were conducted using the intention to treat principle. A p value <0.05 was considered as indication for statistical significance. We did not conduct adjustments for multiple comparisons. All statistical analyses were conducted using SPSS 16.0 for Windows (SPSS, Inc., Chicago, IL.).

## Results

Between august 26^th^ 2,009 and september 1^st^ 2010, thirty patients were randomized. Of these, 14 were randomly assigned to receive aspirin and 16 to no aspirin (table 1). By 2×2 factorial design, 15 patients were randomized to receive atorvastatin and 15 were assigned to no atorvastatin group (table 2). There were no lost during the follow up, but in four patients (one assigned to aspirin, two to atorvastatin and one to no experimental treatment) measurements of TG was not available due to blood samples were not adequate for assays.

**Table 1:** Baseline characteristics of patients randomized to receive aspirin or control.

Variables	Aspirin (n = 14)	Control (n = 16)	p
Age, median (IQR)	56.50 (50.00 to 62.00)	63.00 (58.00 to 71.00)	0.051
Female sex, n (%)	6 (42.9)	8 (53.3)	0.573
Body mass index, mean (SD)	31.97 (6.97)	27.85 (4.87)	0.075
Years since diagnosis, median (IQR)	5.00 (1.00 to 13.25)	9.00 (2.00 to 14.00)	0.505
Blood pressure, mmHg mean (SD)			
Systolic	125.00 (14.01)	132.33 (18.79)	0.247
Diastolic	83.71 (9.61)	82.33 (10.83)	0.720
Heart rate, bpm mean (SD)	75.21 (5.04)	76.93 (7.74)	0.488
Hypertension, n (%)	7 (50)	10 (66.7)	0.362
Smoker, current or former, n (%)	10 (71.4)	8 (53.3)	0.316
HbA1c, % median (IQR)	6.80 (5.83 to 7.20)	6.40 (5.70 to 7.00)	0.621
Plasma glucose, mean (SD)	133.36 (44.57)	120.27 (26.58)	0.341
Diabetes therapy			
Oral hypoglicemic agents, n (%)	11 (78.6)	14 (93.3)	0.330
Metformin	9 (64.3)	10 (66.7)	1.00
Sulfonylurea	6 (42.9)	6 (40.0)	0.876
Insulin, n (%)	4 (28.6)	1 (6.7)	0.169
Total cholesterol, mmol/l median (IQR)	4.9 (4.6 to 5.4)	5.6 (4.7 to 6.1)	0.793
HDL- cholesterol, mmol/l median (IQR)	1.0 (0.9 to 1.4)	1.3 (1 to 1.4)	0.315
LDL- cholesterol, mmol/l median (IQR)	3.1 (2.8 to 3.6)	3.3 (2.5 to 4.0)	0.694
Plasma creatinine, mg/dl mean (SD)	0.77 (0.16)	0.79 (0.13)	0.733
Hematocrit, % mean (SD)	41.40 (4.88)	40.63 (4.74)	0.674
Hemoglobin, gr/dl mean (SD)	13.75 (1.62)	13.33 (1.54)	0.496
Plasma insulin, mean (SD)	12.18 (7.09)	14.55 (5.89)	0.371
HOMA, median (IQR)	4.23 (2.86 to 5.32)	4.47 (3.10 to 5.61)	0.526
C-reactive protein, ng/ml median (IQR)	0.39 (0.08 to 1.6)	0.10 (0.06 to 0.46)	0.218

**Table 2:** Baseline characteristics of patients randomized to receive atorvastatin or control.

Variables	Atorvastatin (n = 15)	Control (n = 15)	p
Age, median (IQR)	62.00 (58.00 to 70.00)	58.50 (50.00 to 65.75)	0.186
Female sex, n (%)	9 (60)	5 (35.7)	0.191
Body mass index, mean (SD)	29.57 (6.14)	30.13 (6.54)	0.813
Years since diagnosis, median (IQR)	5.00 (1.00 to 13.00)	9.50 (3.00 to 22.25)	0.155
Blood pressure, mmHg mean (SD)			
Systolic	127.33 (11.63)	130.36 (21.35)	0.636
Diastolic	82.13 (8.97)	83.93 (11.47)	0.641
Heart rate, bpm mean (SD)	74.40 (6.07)	77.93 (6.71)	0.149
Hypertension, n (%)	8 (53.3)	9 (64.3)	0.550
Smoker, current or former, n (%)	9 (60.0)	9 (64.3)	0.812
HbA1c, median (IQR)	6.70 (5.90 to 6.90)	6.70 (5.68 to 9.13)	0.561
Plasma glucose, mean (SD)	128.87 (34.20)	124.14 (39.64)	0.733
Diabetes therapy			
Oral hypoglicemic agents, n (%)	13 (86.7)	12 (85.7)	1.00
Metformin	9 (60.0)	10 (71.4)	0.700
Sulfonylurea	8 (53.3)	4 (28.6)	0.176
Insulin, n (%)	2 (13.3)	3 (21.4)	0.651
Total cholesterol, mmol/l median (IQR)	4.9 (4.7 to 5.7)	5.1 (4.6 to 6.0)	0.793
HDL- mmol/l median (IQR)	1.3 (1.0 to 1.6)	1.0 (0.9 to 1.3)	0.155
LDL- cholesterol, mmol/l median (IQR)	3.3 (2.5 to 3.9)	3.1 (2.8 to 3.9)	0.647
Plasma creatinine, mg/dl mean (SD)	0.75 (0.16)	0.80 (0.11)	0.317
Hematocrit, % mean (SD)	40.01 (5.45)	42.11 (3.62)	0.250
Hemoglobin, gr/dl mean (SD)	13.23 (1.80)	13.86 (1.23)	0.297
Plasma insulin, mean (SD)	11.86 (5.40)	15.10 (7.32)	0.218
HOMA, median (IQR)	4.47 (3.03 to 5.46)	4.23 (3.10 to 5.86)	0.833
C-reactive protein, ng/ml median (IQR)	0.45 (0.06 to 1.53)	0.15 (0.05 to 0.34)	0.295

Compliance with assigned treatments was high as assessed by pill count and was similar for patients allocated to aspirin and for atorvastatin (>90%). No patient reported the intake of NSAID or any other agent that could modify the results of the TG tests. There were no adverse events during the follow up.

### Baseline data

At baseline, there were no significant differences between treated and control patients in terms of clinical, biochemical, markers of metabolic control and inflammatory parameters (tables 1 and 2).

Overall, the randomized population had good metabolic control as evidenced by mean HbA1 levels in a near optimal range for all patients. Mean fasting plasma LDL-cholesterol levels were between 3.1 to 3.4 mmol/l and C-reactive protein was in the normal range for most of the patients. There was an evenly distributed high prevalence of overweight subjects among groups.

At baseline, all assessments of TG (LT, TTP, PTG and ETP) were similar between treated and control patients (table 3). Similarly, there were no significant differences when diverse agonists were used as ADP, arachidonic acid and tissue factor. For simplicity, we only report the results of PTG with saline solution, tissue factor 1 pM, ADP 8×10^−6^ M, arachidonic acid 25 mmol/L, and for LT with arachidonic acid 25 mmol/L. All other assessments with all other agonists produced similar figures and did not materially change the results and are available upon request. The analyses showed no significant interaction between treatments (p values for PTG with saline solution, tissue factor 1 pM, ADP 8×10^−6^ M and arachidonic acid 25 mmol/L, and for LT with arachidonic acid 25 mmol/L were 0.905, 0.261, 0.711, 0.975 and 0.407, respectively).

**Table 3:** Baseline levels of thrombin generation in patients randomized to aspirin and atorvastatin.

Variables	Aspirin (n = 14)	Control (n = 16)	P value
PTG, nmol/l means (SD) in response to:			
Saline	149.89 (109.68)	276.57 (541.83)	0.868
Tissue factor, 1 pM	289.29 (119.78)	323.51 (176.65)	0.868
ADP, 8×10^−6^ M	186.06 (123.94)	172.61 (98.70)	0.739
Arachidonic acid, 25 nmol/L	186.94 (112.41)	276.65 (466.12)	0.835
LT Arachidonic acid, 25 nmol/L, minutes mean (SD)	7.98 (2.32)	7.02 (1.89)	0.280

	Atorvastatin (n = 15)	Control (n = 15)	
PTG, nmol/l means (SD) generated by:			
Saline	137.79 (109.02)	297.11 (555.70)	0.237
Tissue factor, 1 pM	295.16 (168.68)	319.92 (136.38)	0.419
ADP, 8×10^−6^ M	167.80 (117.39)	189.97 (103.70)	0.443
Arachidonic acid, 25 nmol/L	171.11 (109.26)	298.47 (477.68)	0.548
LT Arachidonic acid, 25 nmol/L, minutes mean (SD)	7.52 (2.41)	7.42 (1.88)	0.901

PTG, peak thrombin generation; LT, lag time.

### Outcomes

At the end of the study, patients assigned to aspirin showed no significant difference in TG as compared with those not receiving aspirin (table 4). Only LT showed a non-significant tendency toward retardation with aspirin (table 4).

**Table 4:** End of the study assessment of thrombin generation in patients randomized to aspirin and in patients randomized to atorvastatin.

Variables	Aspirin (n = 14)	Control (n = 16)	P value
PTG, nmol/l means (SD) in response to:			
Saline	121.51 (81.83)	116.85 (67.66)	0.716
Tissue factor, 1 pM	259.86 (103.89)	257.13 (87.85)	0.833
ADP, 8×10^−6^ M	141.61 (75.55)	160.42 (72.12)	0.703
Arachidonic acid, 25 nmol/L	131.94 (69.43)	139.76 (54.86)	0.828
LT Arachidonic acid, 25 nmol/L, minutes mean (SD)	8.72 (2.27)	7.06 (1.79)	0.055

	Atorvastatin (n = 15)	Control (n = 15)	
PTG, nmol/l means (SD) generated by:			
Saline	85.09 (55.34)	153.26 (75.55)	0.018
Tissue factor, 1 pM	226.62 (93.96)	290.37 (86.51)	0.088
ADP, 8×10^−6^ M	118.49 (54.09)	180.31 (77.40)	0.042
Arachidonic acid, 25 nmol/L	120.35 (51.21)	151.35 (68.70)	0.232
LT Arachidonic acid, 25 nmol/L, minutes mean (SD)	8.73 (2.11)	7.06 (1.97)	0.053

PTG, peak thrombin generation; LT, lag time.

On the other hand, atorvastatin reduced the measures of TG. This reduction was statistically significant for peak assays with saline and ADP 8×10^−6^ M, nearly significant using tissue factor 1 pM, and non-significant using arachidonic acid 25 mmol/L. As occurred with aspirin, trend to increased LT was also noted with treatment with atorvastatin (table 4).

## Discussion

General recommendations for prescription of aspirin among diabetic people are controversial [5]–[8], [10]. Aspirin offers a modest beneficial effect in reducing non-fatal cardiovascular events with no demonstrated benefit on fatal events and a statistically and clinically important increase in bleeding events [5]. In clinical practice, physicians are reluctant to prescribe aspirin in patients with diabetes [9], and at least two large randomized controlled trials [20], [21] not anticipated to report out before 2013 are testing the clinical efficacy of aspirin in people with diabetes. In the meantime, this study provides evidence that statins but not aspirin decrease thrombotic risk among patients with diabetes mellitus. While this was not a “clinical” trial, but rather a pathophysiologic trial, we believe that clinical implications are many and important.

Although the reasons aspirin showed no clear benefit among diabetic patients could reflect a lack of power of individual clinical trials and subgroup analyses, another possibility is that diabetes could represent a special case of aspirin resistance [22]. The poor platelet responsiveness to aspirin – particularly (but not only) the failure in adequately suppressing thromboxane-A2 synthesis – has been proposed as a possible explanation of the failure of antiplatelet therapy to prevent cardiovascular events [22]. Additionally, in the diabetic patient, the antiplatelet effects of aspirin can be overwhelmed by aspirin-insensitive mechanisms of platelet activation and thrombus formation, mainly related to an up-regulated vascular inflammatory reaction [23], [24].

Our results are in line with these reports. In fact, in this population of diabetic patients, aspirin did not decrease the peak levels of TG and failed to reduce the total amount of TG, although, as expected, aspirin delayed the lag time among treated patients. Clinically this could mean that aspirin may be an inefficient/incomplete strategy to reduce thrombotic vascular events in patients with diabetes.

Our results also indicate that the lack of effect of aspirin was independent of the levels of cholesterol as evidenced by the absence of interaction with atorvastatin treatment.

The amount of TG was effectively reduced in the atorvastatin group as compared with the group who did not receive statins. Although most of the effectiveness of statins is related to their ability in reducing plasma lipoproteins, statins also have a variety of effects beyond their lipid lowering actions, including anti-inflammatory and anti-thrombotic actions [25]–[28]. The antithrombotic effects of statins are independent of the cholesterol lowering actions and mostly related to their anti-inflammatory effects [27], [28]. To our knowledge this is the first communication from a randomized trial that demonstrated this important mechanism of statins in patients with diabetes.

It has been shown that diabetes is associated with enhanced inflammatory responses [29] and that there are extensive links between inflammation and hemostatic systems [30]. A number of studies show the response to aspirin is blunted in diabetic patients with high inflammatory marker levels [23], [24], [31]. Our study confirms and extends these observations by showing that an intervention with proven anti-inflammatory effects [27], [28] reduced TG without directly targeting the hemostatic system, giving new insights on the so called “pleiotropic” effects of statins and opening new lines for potential targets in antithrombotic research.

The clinical implications of our findings are important. Although by no means should these results be considered evidence to guide clinical decisions, we believe that the hypothesis is challenging and worth further consideration. Aspirin – as suggested by clinical trials [7], [8] and systematic reviews [4]–[6] – seems to produce neutral/negative results as prevention strategy in people with diabetes. The results of this clinical pharmacology trial confirm and extend this hypothesis.

At the recommended doses used for the prevention of CVD, aspirin does not have any pharmacologic action other than antithrombotic. If the attenuation of those effects demonstrated in this trial is further validated in clinical and basic research, the role of aspirin in diabetic patients will become limited.

In contrast, statins seems to play an important place for prevention of CVD in diabetic patients. Beyond their capacity to reduce cholesterol levels, our results confirm an antithrombotic action of statins, which make them particularly suitable as first line agents to reduce CVD events in people with diabetes.

We believe that some aspects of the methodology used in this trial and the interpretations of the results acknowledge some limitations.

Assessment of previous cardiovascular disease was performed using clinical criteria and a baseline electrocardiogram. Because patients with diabetes may have vascular disease even in the absence of symptoms, instrumental screening tests may provide additional information. However, even in the case that some patient may had silent CAD, this would not change conceptually our results.

Considering that methods to assess TG are not fully standardized, it would have been helpful to additionally study a control group of non diabetic patients as controls. Although this would have yielded additional robustness to the method, the clinical implications of the obtained results would not change.

As we previously stated, our results by no means should guide clinical decisions. Since this is not a clinical but rather a pharmacologic clinical trial, the results should be interpreted with caution. Aspirin may reduce thrombotic risk by mechanisms not measured in TG assay. Although this is possible, our results extends previous, population, clinical and basic data on the limited value of aspirin as a general strategy for people with diabetes and no previous cardiovascular events.

Although TG is a valid and interesting method to assess many aspects of thrombotic risk, is should be noted –however- that this is a physiologic and not a clinical end point. Moreover, TG is not part of normal/usual assays of normal clinical practice and so far, has mainly academic/research interest. Many aspects of TG, including stability, optimal agonists and optimal doses of agonists lack of universal agreement. Despite this, our results are consistent with all different agonists at different doses, in plasma-rich-platelets as well as in plasma-poor-platelets. In this sense, our results tested and suggested a proof of concept (i.e. experimental and specific assessment of thrombotic risk confirms and extends previous observation on the limited value of aspirin as an universal prevention strategy in patients with diabetes and the appealing role of statins).

Finally, TG is associated with venous thrombotic risk [13] and its role with arterial thrombotic risk is less well established. A recent communication [32] found that elevated TG was an independent risk factor for acute ischemic stroke but failed to demonstrate a statistical significant association with coronary artery disease. Although the association between TG and arterial vascular risk is documented [32], extrapolate the results to all vascular territories is not yet guarantee.

## Supporting Information

Protocol S1 **Trial protocol.**
(DOC)

Checklist S1 **CONSORT Checklist.**
(DOC)
