# Supplementary material for: Statins but Not Aspirin Reduce Thrombotic Risk Assessed by Thrombin Generation in Diabetic Patients without Cardiovascular Events: The RATIONAL Trial
Source: PLoS One. 2012 Mar 28;7(3):e32894. doi: 10.1371/journal.pone.0032894 (PMC3314656; doi:10.1371/journal.pone.0032894)
Supplement: Protocol S1 — Trial protocol. (DOC) [file pone.0032894.s001.doc]

## Protocolo

EVALUACION DE LA EFICACIA DE ASPIRINA, ESTATINAS O AMBAS PARA LA REDUCCION DE LA GENERACION DE TROMBINA Y MARCADORES INFLAMATORIOS EN PACIENTES diabéticos SIN EVENTOS CARDIOVASCULARES PREVIOS

(AspiRin StAtins Or BoTh for the ReductIon of ThrOmbin GeNeration In DiAbetic PeopLe. The RATIONAL Study)

# Versión 3: 01 de Junio de 2009

**Introducción**

Está ampliamente documentado que la aspirina (AAS) resulta útil para la prevención de eventos vasculares mayores en pacientes que han sufrido eventos cardiovasculares previos (prevención secundaria). Por otro lado, en sujetos sin antecedentes de eventos cardiovasculares (prevención primaria), la AAS se demostró efectiva en determinados grupos de riesgo en donde la evidencia de beneficio (reducción de los eventos vasculares, particularmente infarto de miocardio) era mayor que la de daño (sangrado mayor intra y extracraneano) (1-5).

Estos mismos análisis demostraron también que la AAS no disminuyó en forma significativa la tasa de eventos vasculares en pacientes con diabetes mellitus. Particularmente en este grupo la reducción relativa de riesgo de eventos vasculares mayores fue de un no significativo 9% contra una significativa reducción de 22% en todos los demás grupos sin diabetes (4). Estos resultados derivan básicamente del estudio ETDRS (Early Treatment Diabetic Retinopathy Study), el único que involucró un número importante de pacientes diabéticos (n=3,711), en el cual la aspirina se asoció a un efecto neutro tanto sobre la mortalidad como sobre los eventos cardiovasculares (6). Mas aún, esta aparente ausencia de beneficio no se limita solo a la prevención secundaria. Un análisis de subgrupos de mas de 1,000 pacientes diabéticos en el PPP (Primary Prevention Project) mostró que la aspirina no modificó el riesgo de eventos cardiovasculares mayores en el seguimiento a 3 años (Riesgo relativo = 0.90) (7,8).

A pesar de la actual recomendación de la “Asociación Americana de Diabetes” para el uso de aspirina en pacientes diabéticos (9), los resultados de los ensayos clínicos y meta-análisis, además de no ser concluyentes, sugieren que éste grupo de pacientes podría ser considerado “no respondedor” a la aspirina. La implicancia de esta hipótesis tiene particular interés en salud pública. Si, efectivamente la aspirina resultara ineficaz en pacientes diabéticos, rapidamente deberían revisarse las lineas guias y recomendaciones, ya que –como se expuso en forma precedente- el uso del ácido acetilsalicílico se asocia a un aumento del riesgo relativo de sangrado craneano de 32% y de sangrado extracranial de 55%.

Los mecanismos de esta falta de respuesta no están del todo claros, pero varias líneas de investigación apoyan al menos 2 factores principales: el estado inflamatorio activado que lleva a la activación plaquetaria y a una activación pro-trombótica por vías diferentes de la del tromboxano, y el fracaso de la aspirina para suprimir completamente la producción de TxA2 en pacientes diabéticos (10).

La enfermedad vascular de los diabéticos se caracteriza por una progresión acelerada de la aterosclerosis (11), acompañada de una incidencia incrementada de complicaciones como ulceración, fisura y trombosis de la placa aterosclerótica. Mas aún, los fenómenos inflamatorios parecen estar mas presentes en la placa aterosclerótica de los pacientes diabéticos que en la de los no diabéticos (12). Factores específicos presentes en diabéticos como la hiperglucemia, la hiperinsulinemia, el estrés oxidativo aumentado y una mayor glicosilación de productos finales pueden “up-regular” una variedad de reacciones inflamatorias – humorales, celulares y particularmente trombogénicas, que juegan un rol importante en la iniciación, progresión y precipitación de la aterosclerosis (13-20). Estos hallazgos sugieren que el fracaso clínico de la aspirina en diabéticos podría deberse a mecanismos no relacionados con su principal efecto bioquímico en su blanco farmacológico - la síntesis de tromboxano A2 y la activación plaquetaria - y subraya los aspectos inflamatorios y trombóticos subyacentes como las principales causas de resistencia.

Por otro lado, estudios recientes documentaron que en algunos pacientes tratados con aspirina, el riesgo de experimentar un nuevo evento cardiovascular mayor se correlacionaba con una reducida capacidad de la aspirina para inhibir adecuadamente la síntesis de Tromboxano A2 *in vivo* (21), así como también para disminuir adecuadamente la activación y la agregación plaquetaria *ex vivo* (22). Esto sugiere que, al menos en subpoblaciones de pacientes de alto riesgo cardiovascular, los mecanismos directamente relacionados a la menor capacidad de la aspirina para alterar la síntesis de TxA2 y afectar la función plaquetaria, pueden ser responsables de este fracaso de la aspirina. De hecho, la síntesis de TxA2 y la función plaquetaria se encuentran up-reguladas en pacientes diabéticos e incluso aceleradas por la hiperglucemia aguda (23). Se ha sugerido además, que la eficacia de la aspirina para inhibir la función plaquetaria ex vivo podría ser mas baja en pacientes diabéticos (24-25).

En conclusión, los factores inflamatorios y trombogénicos extra-plaquetarios tanto como los mecanismos intraplaquetarios, pueden contribuir a la compleja falla de la aspirina en los diabéticos.

Las estatinas, por su parte, poseen efectos beneficiosos tanto en prevención primaria como secundaria en la enfermedad coronaria (26). Una serie de efectos pleiotrópicos (27), así como la precocidad de su acción terapéutica (28), sugieren que su beneficio va mas allá de la mera reducción de los valores del colesterol e involucra un efecto anti-inflamatorio y antitrombotico. En contraste con la aspirina, la magnitud de los beneficios obtenidos con estatinas es similar en pacientes diabéticos y no diabéticos (29), sugiriendo que los mecanismos anti-inflamatorios modificados por las estatinas son diferentes y complementarios a aquellos de la aspirina. Por su parte, plaquetas obtenidas de pacientes hiperlipidémicos son altamente trombogénicas, hecho que fue asociado a una peor respuesta plaquetaria a la aspirina (30).

Recientemente (31) se documentó que la aspirina tiene una moderada acción antitrombótica directa, disminuyendo los niveles de trombina plasmática en aproximadamente un 20 - 40% (32-41). Aún más interesante resulta el hecho que la acción antitrombótica de la aspirina parece ser dependiente de los niveles plasmáticos de colesterol (42). En este sentido la aspirina solo parecería tener acción antitrombótica cuando los niveles de colesterol plasmático no son elevados (42). Las estatinas también tienen acción antitrombótica directa y el efecto parece no tener solo relación con su efecto hipolipemiante sino sobretodo por su mecanismo anti-inflamatorio (31).

Curiosamente, a pesar de lo mencionado, no existen estudios que examinen la efectividad de cada una de estas drogas, solas o en combinación, en pacientes diabéticos.

En resumen,

- No existe evidencia directa concluyente sobre la eficacia de la AAS en pacientes con diabetes.
- Diversos mecanismos fisiopatológicos presentes en la diabetes podrían justificar un efecto reducido de la AAS.
- Dos ensayos clínicos de grandes dimensiones y actualmente en marcha evalúan este tópico. Sin embargo los resultados estarán disponibles solo a fines del año 2012.
- Las estatinas son igualmente útiles en pacientes con y sin diabetes.
- El efecto de las estatinas está más allá del efecto hipolipemiante e involucra mecanismos anti inflamatorios y antitrombóticos.
- Pese a esto, no existen trabajos que especifícamente examinen si la asociación de estatinas con aspirina en pacientes diabéticos, resultan superiores al solo uso de cada uno de estos agentes.
- En el estado actual de conocimientos probablemente la propuesta de “aspirina a todos los diabéticos sin eventos cardiovasculates previos por toda la vida”, no está sustentada suficientemente y hasta podria resultar en un exceso de eventos hemorrágicos.

Se propone realizar un ensayo clínico de pequeñas dimensiones, con puntos finales surrogados, de naturaleza exploratoria desde el punto de vista fisiopatológico, para evaluar si la asociación de aspirinas y estatinas resultan mas efectivas que cada uno de estos agentes para la disminución de los niveles en plasmáticos de trombina, que constituye el efector final de la interacción inflamación-trombosis.

**PACIENTES Y METODOS**

El ensayo clínico que se propone es de tipo pragmático con la intención que se asemeje en la mayor medida de lo posible a la práctica clínica cotidiana, con el doble propósito de hacerlo transferible a contextos asistenciales reales y con el objetivo de impactar poco en los costos de la asistencia.

Se incluirán en este estudio pacientes diabéticos tipo II sin antecedentes cardiovasculares previos y que al momento de la firma del consentimiento informado no estaban recibiendo tratamiento con aspirina, ni estatinas ni tenian indicación -a criterio de su médico tratante- de recibirlas (ver criterios de inclusión y exclusión).

### DISEÑO

Estudio prospectivo, randomizado, abierto, ciego a las determinaciones (diseño PROBE) con asignación aleatoria y centralizada a:

Rama 1: Control (los pacientes continúaran durante la duración del estudio planificada en 8 semanas sin recibir ni aspirina ni estatinas como lo estaban haciendo hasta el momento de la firma del consentimiento informado).

Rama 2: Solo Aspirina (100 mg / día de AAS)

Rama 3: Solo Estatinas (40 mg / día de Atorvastatina)

Rama 4: Tratamiento Combinado (AAS 100 mg /dia + Atorvastatina 10 mg / dia)

Los pacientes serán randomizados en forma central. En caso que el estudio involucre a mas de un centro asistencial, la randomización será estratificada por centro.

El estudio está diseñado para ser realizado en centros que cuenten con consultorios externos y con posibilidad de realizar extracciones de sangre periférica en forma ambulatoria, sin la necesidad de instalaciones especiales. El período de inclusión será de aproximadamente 6 meses y la duración de la intervención de 8 semanas.

### POBLACION

La población del estudio estará constituida por personas de ambos sexos, en seguimiento ambulatorio con diabetes mellitus tipo II y que además presenten todos los criterios de inclusión y ninguno de los de exclusión que se enumeran a continuación.

#### Criterios de Inclusión

Al momento de la firma del consentimiento informado, todos los pacientes deben presentar las siguientes cuatro características:

1) Tratamiento actual con hipoglucemiantes orales y/o insulina desde al menos 6 meses antes de la firma del consentimiento informado por diagnóstico de diabetes mellitus tipo II.

2) No estar en tratamiento actual ni haber recibido tratamiento previo (en los 6 meses precedentes a la firma del consentimiento informado) con estatinas

3) No estar en tratamiento actual ni haber recibido tratamiento previo (en el mes precedente a la firma del consentimiento informado) con aspirina

4) Edad ≥ 50 años

#### Criterios de exclusión

1) Edad < 50 años

2) Evento cardiovascular previo (infarto de miocardio, angina, revascularización o test funcional positivo) según conste en la documentación clínica en la historia clínica del paciente.

3) Cualquier condición que, a juicio del investigador, pudiera perjudicar la evaluación de la eficacia o seguridad del protocolo, o afectara la adherencia al mismo.

4) Tratamiento actual con estatinas y/o aspirina (ver criterios de inclusión) o indicación según el criterio del médico tratante de recibirlas.

5) Tratamiento actual o previo (≤ 1 mes) con cualquier agente anti-inflamatorio no esteroideo, clopidogrel, ticlopidina, dipiridamol, anticoagulación oral (tanto con acenocumarol o warfarina).

6) Procedimientos quirúrgicos previos (≤ 1 mes) o planificados al momento de la firma del consentimiento informado (para los próximos 3 meses desde la fecha de firma del consentimiento informado).

7) Incapacidad para entender o firmar el consentimiento informado.

### TRATAMIENTO

#### Drogas de investigación y control

Las siguientes drogas de estudio serán administradas:

Aspirina 100 mg diarios en una toma.

Atorvastatina 40 mg diarios en una toma.

Combinación (en comprimidos separados) de Aspirina 100 mg diarios y Atorvastatina 40 mg en una toma.

#### Grupos de tratamiento

Los pacientes serán asignados a recibir una de las 4 estrategias propuestas y seguirán este tratamiento hasta finalizar el estudio. La duración del tratamiento será de 8 semanas desde la randomización.

#### Otros tratamientos concomitantes

Todos los tratamientos indicados para la diabetes serán permitidos. Una dieta adecuada, actividad física y otras actividades higiénico-dietéticas serán fuertemente recomendadas de acuerdo a las guías de práctica clínica vigentes.

El investigador deberá instruir al paciente a notificar al centro coordinador de cualquier nueva medicación que sea iniciada luego del inicio de la droga del estudio. Todas las medicaciones concomitantes serán registradas.

#### Discontinuación de la droga

La discontinuación de la medicación del estudio será considerada (y mientras sea posible discutida con el Centro Coordinador) sólo si una de las siguientes condiciones está presente:

a) El paciente decide retirar su consentimiento a continuar participando del estudio. A partir de ese momento no continuará recibiendo la medicación del estudio ni ningún otro dato será recolectado en el CRF;

b) El investigador considera que es aconsejable debido a una razón clínica relevante, explícita y documentada;

c) Un evento adverso serio (incluyendo alteraciones de laboratorio) que se sospeche que pueda estar relacionado con la medicación del estudio y/o evite que pueda continuarse con la medicación del estudio.

**PUNTO PRIMARIO DE EFICACIA.**

El punto final de eficacia de este ensayo clínico será la determinación de los niveles de generación de trombina al momento de finalización del estudio y su comparación con los niveles basales.

**OTROS PUNTOS DE EFICACIA.**

En todos los pacientes se evaluarán además una serie de marcadores inflamatorios inmediatamente posterior a la randomizacion y al finalizar el estudio, incluyendo los niveles de proteina C reactiva y SCD40L, sP-selectina, sICAM-1, sE-selectina y factor tisular serico.

### PLAN ESTADISTICO

Objetivo primario de eficacia: En pacientes diabéticos los valores medios de trombina medida por el método de Hemker y col.con el uso del CAT (Calibrated Automated Thrombogram) es de 150 nM ± 45 nM.

Diversas comunicaciones reportan que la AAS disminuye los niveles de trombina en aproximadamente 30%. En consecuencia el número necesario de sujetos para demostrar una reducción con AAS de 30% con un poder de 0.95% y un error alfa de 0.05 es de 13 sujetos en la rama con solo aspirina.

Para poder demostrar la hipótesis que el agregado de estatinas disminuye ulteriormente un 30% adicional los niveles de trombina (una reducción de 60% con respecto a los niveles basales) en la rama de tratamiento combinado harían falta solo 4 pacientes en este grupo.

Considerando que se usará como grupo control un número de sujetos similares a la numerosidad de los otros grupos se conformaran 4 grupos de 20 pacientes cada uno.

Las SCD40L, sP-selectina, sICAM-1 y sE-selectina serán medidas en plasma mediante kits comerciales Elisa (todos ellos de Bender Med Systems, Viena - Austria). El factor tisular sérico será medido en plasma utilizando kits Elisa de American Diagnostica (Instrumentation Laboratory, Milan, Italia).

Mas allá de todas las determinaciones relacionadas con el presente protocolo de investigación, los marcadores tradicionales de glucemia, perfil lipídico y microalbuminuria serán medidos en el laboratorio central utilizando los métodos estándar en todos los pacientes. La glucosa plasmática en ayunas será medida mediante el método oxidativo. El colesterol total y los triglicéridos, mediante el método enzimático; la lipoproteína de alta densidad (HDL) será medida en suero luego de precipitada en ácido fosfotúngstico/MgCI2. La fórmula de Friedwald se utilizará par calcular el colesterol LDL. Las concentraciones urinarias de albúmina serán determinadas mediante el método inmune turbidimétrico (albumin-tinaquant, Roche Diagnostics). A todos los pacientes se les realizará una determinación de urea, creatinina, TGO, TGP y LDH al momento del inicio del estudio y al finalizar el mismo. Asimismo al iniciar y finalizar el estudio se medira en todos los pacientes los niveles de HOMA (Homeostasis Model Assessment) y proteina C reactiva.

### BUENAS PRACTICAS CLINICAS

La última revisión de la declaración de Helsinki y también de la declaración de Oviedo proveen el marco general para los aspectos éticos de este estudio. El protocolo del estudio está diseñado para asegurar adherencia a los principios y procedimientos de buenas prácticas clínicas (GCP), como están descriptos en los siguientes documentos:

Guías ICH Armonizadas de Buenas Prácticas Clínicas; Directiva 91/507/EEC, “The Rules Governing Medicinal Products in the European Community”; US 21 Code of Federal Regulations, tratando los ensayos clínicos (incluyendo partes 50 y 56 sobre el consentimiento informado y regulación de comités de ética) y la disposición 5330/97 ANMAT con sus modificaciones.

Comité de ética independiente/Comités de revisión institucionales (CEI/CRI)

Antes de implementar el estudio, el consentimiento informado propuesto y otras informaciones para pacientes debe ser revisado por un CEI/CRI apropiadamente constituido. Una declaración escrita, fechada y firmada que el protocolo y el consentimiento informado han sido aprobados por el CEI/CRI debe ser dado al Centro Coordinador antes del inicio del estudio.

#### Consentimiento informado

El Centro Coordinador proveerá una propuesta de consentimiento informado, que sea parte del protocolo y cumpla con los requerimientos regulatorios deberá ser aprobado por el CEI/CRI junto con el protocolo.

#### Seguro

El estudio será asegurado con una póliza de seguros dotada de los montos adecuados a la relevancia del mismo. Se enviará al Comité de Etica una copia de la citada póliza

#### Randomización

Todos los pacientes que cumplan con los criterios de inclusión y exclusión serán asignados en forma central con un número de randomización. Para obtener el número de randomización un procedimiento de randomización centralizado telefónico será implementado. Los siguientes datos serán requeridos al momento de la randomización:

· Fecha de firma del consentimiento informado

· Iniciales del paciente

· Fecha de nacimiento

· Sexo

· Cumplimiento de los criterios de selección

EL sistema solicitará confirmar los criterios de selección. El paciente será considerado randomizado cuando el sistema le asigne un número de randomización y tratamiento. Este número será consignado en el formulario (CRF) en la sección de inclusión.

El investigador recibirá confirmación de la inclusión del paciente con el número de randomización y tratamiento inicialmente por fax, firmada por un responsable del centro coordinador.

#### Evaluación de pacientes y visitas de seguimiento

Visita 0 (Screening): El paciente será invitado a participar del estudio y confirmará su libre voluntad de participar mediante la firma del consentimiento informado. El paciente puede firmarlo en ese momento o solicitar una nueva visita para comunicar su decisión y eventualmente firmarlo.

Visita 1: Se realizará la randomización, historia clínica, examen físico, signos vitales, entrega de medicación, revisión de medicación del estudio y concomitante. Se completará el CRF y se solicitará el laboratorio inicial según descripto precedentemente

Visita 2 ó de Finalización del Estudio: Esta visita se realizará a las 8 semanas o ante la finalización de la participación del paciente en el estudio por cualquier motivo. Se realizará examen físico, signos vitales, análisis de laboratorio, registro de eventos adversos, e información de finalización del estudio. Al recibir los resultados de laboratorio el paciente será contactado telefónicamente para reportarle los resultados.

El investigador deberá instruir al paciente a tomar la medicación y se procurará mantener elevado el nivel de adherencia.

#### Seguridad

Debido a que el presente ensayo clínco a) no evalúa nueva indicación y b) tiene una duración de sólo 8 semanas, no se realizará análisis interino de seguridad, aunque sí se procederá por parte del investigador principal a un estricto control de los eventos adversos, según se comunica en la siguiente sección.

#### Eventos adversos (EA)

Un evento adverso es la aparición o empeoramiento de cualquier signo, síntoma o condición no deseada que se inicie luego del inicio de la medicación del estudio, aun si el evento no es considerado como relacionado con la medicación del estudio. La medicación del estudio incluye todas las drogas en evaluación. Las condiciones médicas o enfermedades presentes antes del inicio de la medicación del estudio sólo se consideran eventos adversos si empeoran luego del inicio de la medicación. Resultados anormales de laboratorio constituyen eventos adversos si inducen signos o síntomas, son considerados clínicamente significativos o requieren tratamiento.

Todos los EA se reportarán a los comités de ética desde la firma del consentimiento.

Cada evento adverso será evaluado para determinar:

1. su relación con la(s) droga(s) del estudio (sospechada o no sospechada)

2. su duración (fechas de inicio y fin o si continúa al final del estudio)

3. acción llevada a cabo (ninguna; interrupción temporaria; interrupción permanente; medicación concomitante indicada; tratamiento no farmacológico; hospitalización)

4. si es o no serio

Todos los eventos adversos deben ser tratados apropiadamente. El tratamiento incluye cambios en la medicación del estudio (interrupción temporaria o permanente), inicio o suspensión de otras medicaciones concomitantes, cambios en la frecuencia o tipo de evaluaciones, hospitalización o cualquier otra intervención médica requerida. Una vez que un evento adverso es detectado, debe ser seguido hasta su resolución.

Eventos adversos serios (SAEs)

Un evento adverso serio se define como

· Es fatal o amenaza la vida

· Produce incapacidad persistente o significativa

· Requiere hospitalización o prolongación de una hospitalización, excepto que la hospitalización sea por:

a) tratamiento de rutina o monitoreo de la indicación en estudio, no asociado con empeoramiento de la condición

b) tratamiento electivo o pre-planificado de una condición preexistente que no está relacionada a la indicación en estudio y no se ha empeorado desde el inicio del tratamiento en estudio.

c) tratamiento ambulatorio no programado de un evento no cumpliendo con los criterios de un SAE definidos arriba y no resultando en hospitalización.

d) razones sociales en ausencia de deterioro del estado clínico

· Es médicamente significativo: definido como un evento que amenaza al paciente o puede requerir intervención médica para prevenir alguno de los resultados descriptos más arriba.

#### Comités del estudio

El Comité de Dirección (Steering committee) actuará como patrocinante del estudio y tiene completa responsabilidad de planificación, conducción, análisis y publicación de resultados. Este comité está compuesto por los doctores Alejandro Macchia, Alejandra Scazziotta, Roberto Altman, Cristián López Saubidet.

#### Registro de datos y retención de documentos

Los documentos esenciales, listados debajo, deben ser retenidos por el investigador tanto tiempo como sea necesario para cumplir con las regulaciones nacionales e internacionales (en general 2 años luego del desarrollo clínico o de la última aprobación de mercado). El personal del estudio notificará a los centros cuando los registros del estudio no sean ya requeridos.

El investigador acuerda adherir a los procedimientos de retención de documentos al firmar el protocolo. Los documentos esenciales incluyen:

· Aprobaciones de comités de ética del protocolo del estudio y sus enmiendas

· Documentos fuente y registros de laboratorio

· Copias de CRF

· Consentimientos informados de los pacientes

· Cualquier otro documento pertinente

#### Procedimientos de auditoría

Se esperará la realización de inspecciones por autoridades regulatorias, y serán bienvenidas.

#### Recolección de datos

Los investigadores deberán consignar la información requerida en el CRF. Los CRFs serán enviados al Centro Coordinador. Una copia del CRF será retenida en el centro. Una vez que los CRFs son recibidos, se registrará su recepción, y serán entregados al personal encargado del manejo de datos para su procesamiento.

#### Política de publicación

La publicación de los resultados de eficacia como están definidos en el protocolo serán publicados por el Comité de Dirección, siendo además facultad de este Comité decidir incluir otros autores según se disponga en reuniones que se realizarán oportunamente y finalizado el estudio.

Un apéndice incluirá los nombres de los representantes de cada centro participante.

REFERENCIAS

1- Antiplatelet Trialists' Collaboration. Collaborative overview of randomised trials of antiplatelet therapy. I. Prevention of death, myocardial infarction, and stroke by prolonged antiplatelet therapy in various categories of patients. BMJ 1994;308:81106.

2- Antiplatelet Trialists' Collaboration. Collaborative overview of randomised trials of antiplatelet therapy. II. Maintenance of vascular graft or arterial patency by antiplatelet therapy. BMJ 1994;308:15968.

3- Antiplatelet Trialists' Collaboration. Collaborative overview of randomised trials of antiplatelet therapy. III. Reduction in venous thrombosis and pulmonary embolism by antiplatelet prophylaxis among surgical and medical patients. BMJ 1994;308:23546.

4- Antiplatelet Trialists' Collaboration. Collaborative meta-analysis of randomised trials of antiplatelet therapy for prevention of death, myocardial infarction, and stroke in high-risk patients. BMJ 2002;324:7186.

5- Eidelman RS, Herbert PR, Weisman SM, Hennekens CH. An update on the primary prevention of cardiovascular disease. Arch Intern Med. 2003;163:2006-2010.

6- ETDRS Investigators. Aspirin effects on mortality and morbidity in patients with diabetes mellitus. Early treatment diabetic retinopathy study report 14. JAMA 1992;268:1292300.

7- Collaborative Group of the Primary Prevention Project (PPP). Low-dose aspirin and vitamin E in people at cardiovascular risk: a randomised trial in general practice. Collaborative Group of the Primary Prevention Project. Lancet 2001;357:89-95.

8- Sacco M, Pellegrini F, Roncaglioni MC, Avanzini F, Tognoni G, Nicolucci A; PPP Collaborative Group. Primary prevention of cardiovascular events with low-dose aspirin and vitamin E in type 2 diabetic patients: results of the Primary Prevention Project (PPP) trial. Diabetes Care. 2003;26:3264-72.

9- Colwell JA, American Diabetes Association. Aspirin therapy in diabetes. Diabetes Care 2003; 26:S87-S88

10- Evangelista V, Totani L, Rotondo S, Lorenzet R, Tognoni G, De Berardis G, Nicolucci A. Prevention of cardiovascular disease in type-2 diabetes: how to improve the clinical efficacy of aspirin. Thromb Haemost 2005; 93:8-16

11- Dortimer AC, Shenoy PN, Shiroff RA, Leaman DM, Babb JD, Liedtke AJ, Zelis R. Diffuse coronary artery disease in diabetic patients: fact or fiction? Circulation. 1978;57:133–136.

12- Moreno PR, Murcia AM, Palacios IF, Leon MN, Bernardi VH, Fuster V, Fallon JT. Coronary composition and macrophage infiltration in atherectomy specimens from patients with diabetes mellitus. Circulation. 2000;102:2180–2184.

13- Ceriello A. Coagulation activation in diabetes mellitus: the role of hyperglycaemia and therapeutic prospects. Diabetologia. 1993;36:1119–1125.

14- Rao AK, Chouhan V, Chen X, Sun L, Boden G. Activation of the tissue factor pathway of blood coagulation during prolonged hyperglycemia in young healthy men. Diabetes. 1999;48:1156–1161.

15- Takahashi T, Hato F, Yamane T, Inaba M, Okuno Y, Nishizawa Y, Kitagawa S. Increased spontaneous adherence of neutrophils from type 2 diabetic patients with overt proteinuria: possible role of the progression of diabetic nephropathy. Diabetes Care. 2000;23:417–418.

16- Shurtz-Swirski R, Sela S, Herskovits AT, Shasha SM, Shapiro G, Nasser L, Kristal B. Involvement of peripheral polymorphonuclear leukocytes in oxidative stress and inflammation in type 2 diabetic patients. Diabetes Care. 2001;24:104–110.

17- Koyama T, Nishida K, Ohdama S, Sawada M, Murakami N, Hirosawa S, Kuriyama R, Matsuzawa K, Hasegawa R, Aoki N. Determination of plasma tissue factor antigen and its clinical significance. Br J Haematol. 1994;87:343–347.

18- Jude B, Watel A, Fontaine O, Fontaine P, Cosson A. Distinctive features of procoagulant response of monocytes from diabetic patients. Haemostasis. 1989;19:65–73.

19- Diamant M, Nieuwland R, Pablo RF, Sturk A, Smit JW, Radder JK. Elevated numbers of tissue-factor exposing microparticles correlate with components of the metabolic syndrome in uncomplicated type 2 diabetes mellitus. Circulation. 2002;106:2442–2447.

20- Rauch U, Crandall J, Osende JI, Fallon JT, Chesebro JH, Fuster V, Badimon JJ. Increased thrombus formation relates to ambient blood glucose and leukocyte count in diabetes mellitus type 2. Am J Cardiol. 2000;86:246–249.

21- Eikelboom JW, Hirsh J, Weitz JI, Johnston M, Yi Q, Yusuf S. Aspirin-resistant thromboxane biosynthesis and the risk of myocardial infarction, stroke, or cardiovascular death in patients at high risk for cardiovascular events. Circulation. 2002;105:1650–1655.

22- Gum PA, Kottke-Marchant K, Poggio ED, Gurm H, Welsh PA, Brooks L, Sapp SK, Topol EJ. Profile and prevalence of aspirin resistance in patients with cardiovascular disease. Am J Cardiol. 2001;88:230–235.

23- Gresele P, Guglielmini G, De Angelis M, Ciferri S, Ciofetta M, Falcinelli E, Lalli C, Ciabattoni G, Davi G, Bolli GB. Acute, short-term hyperglycemia enhances shear stress-induced platelet activation in patients with type II diabetes mellitus. J Am Coll Cardiol. 2003;41:1013–1020.

24- Watala C, Golanski J, Pluta J, Boncler M, Rozalski M, Luzak B, Kropiwnicka A, Drzewoski J. Reduced sensitivity of platelets from type 2 diabetic patients to acetylsalicylic acid (aspirin)-its relation to metabolic control. Thromb Res. 2004;113:101–113.

25- Fateh-Moghadam S, Plockinger U, Cabeza N, Htun P, Reuter T, Ersel S, Gawaz M, Dietz R, Bocksch W. Prevalence of aspirin resistance in patients with type 2 diabetes. Acta Diabetol. 2005;42:99–103.

26- Law MR, Wald NJ, Rudnicka AR. Quantifying effect of statins on low density lipoprotein cholesterol, ischaemic heart disease, and stroke: systematic review and meta-analysis. BMJ. 2003;326:1423

27- Davignon J. Beneficial cardiovascular pleiotropic effects of statins. Circulation 2004;109 Suppl 1):III39-43

28- Ray KK, Cannon CP. Early time to benefit with intensive statin treatment: could it be the pleiotropic effects? Am J Cardiol. 2005;96(5A):54F-60F.

29- Collins R, Armitage J, Parish S, Sleigh P, Peto R: MRC/BHF Heart Protection Study of cholesterol-lowering with simvastatin in 5963 people with diabetes: a randomised placebo-controlled trial. Lancet 361: 2005–2016, 2003.

30- Friend M, Vucenik I, Miller M. Platelet responsiveness to aspirin in patients with hyperlipidemia. BMJ 2003;326:82-83.

31 – Undas A. Simvastatin Depresses Blood Clotting by Inhibiting Activation of Prothrombin, Factor V, and Factor XIII and by Enhancing Factor Va Inactivation. Blood 2007;109:2285-92

32- Kyrle PA Investigation of the interaction of blood platelets with the coagulation system at the site of plug formation ex vivo in man: effect of low-dose AAS. Thromb Haemost 1987; 57: 62–69.

33- Hampton KK. Coagulation, fibrinolytic and platelet function in patients in long-term therapy with 300 mg or 1200 mg daily compared with placebo. Thromb Haemost

1990; 64: 17–24.

34 – Szczeklik A. Antiplatelet drugs and generation of thrombin in clotting blood Blood. 1992;80:2006-2011.

35 - Kessels H Measurement of thrombin generation in whole blood--the effect of heparin and AAS. Thromb Haemost 1994; 72: 78–83.

36 - Reverter JC. Inhibition of platelet-mediated, tissue factor-induced thrombin generation. J Clin Invest 1996; 98: 863–74.

37 - Musial J. Aspirin delays thrombin generation in vitro through interaction with platelet phospholipids. Thromb Res 1997; 85: 367–8.

38 - Wallen NH. Influence of low- and high-dose aspirin treatment on thrombin generation in whole blood. Thromb Res 1997; 85: 367–8.

39 - Undas A A low dose of AAS (75 mg/day) lowers thrombin generation to a similar extent as a high dose of AAS (300 mg/day) Blood Coagul Fibrinolysis. 2000;11:231-4.

40 - Undas A Blood coagulation at the site of microvascular injury: effects of low dose aspirin. Blood 2001; 98:2423-31.

41 - Hemker HC. Calibrated automated thrombin generation measurement in clotting plasma. Pathophysiol Haemost Thromb. 2003;33:4-15.

42 - Szczeklik A. Inhibition of thrombin generation by aspirin is blunted in hypercholesterolemia. Arterioscler Thromb Vasc Biol 1996;16:948-54
